# Prevalence of malocclusion in Turkish children and adolescents: A systematic review and meta‐analysis

**DOI:** 10.1002/cre2.771

**Published:** 2023-08-14

**Authors:** Jimmy Londono, Shohreh Ghasemi, Negar Moghaddasi, Homa Baninajarian, Amir Fahimipour, Sara Hashemi, Amirhossein Fathi, Mahmood Dashti

**Affiliations:** ^1^ Director of the Prosthodontics Residency Program and the Ronald Goldstein Center for Esthetics and Implant Dentistry Augusta Georgia USA; ^2^ Department of Oral and Maxillofacial Surgery The Dental College of Georgia at Augusta University Augusta Georgia USA; ^3^ DDS, College of Dental Medicine Western University of Health Sciences California USA; ^4^ Dental Research Center Isfahan University of Medical Sciences Isfahan Iran; ^5^ Discipline of Oral Surgery, Medicine and Diagnostics, School of Dentistry, Faculty of Medicine and Health, Westmead Centre for Oral Health The University of Sydney New South Wales Australia; ^6^ Dental Students' Research Committee Isfahan University of Medical Sciences Isfahan Iran; ^7^ Dental Prosthodontics Department, Dental Materials Research Center Isfahan University of Medical Sciences Isfahan Iran; ^8^ School of Dentistry Shahid Beheshti University of Medical Sciences Tehran Iran

**Keywords:** epidemiology, malocclusion, prevalence

## Abstract

**Objectives:**

The aim of this article is to establish a comprehensive nationwide prevalence of malocclusion traits on the sagittal, vertical, and transverse planes of space in the Turkish population.

**Material and Methods:**

A systematic search of PubMed, Scopus, and Web of Science was supplemented by manual searches of Google Scholar and the reference lists of included studies. Original Turkish health studies of any age were included. Strengthening the Reporting of Observational Studies in Epidemiology assessed study quality and bias (STROBE). Sagittal, vertical, and transverse malocclusion features were retrieved and gathered.

**Results:**

Eleven studies were selected from 434 titles. Two studies showed a high risk of bias, eight low and one moderate. Thirteen thousand two hundred seventy‐one individuals were investigated from early childhood to late adulthood. Most studies were sampled from universities and dental (nonorthodontic) clinics. The pooled malocclusion prevalence was 56% for Class I (95% confidence interval (CI): 44−68%), 31% for Class II (CI: 6–42%), and 11% for Class III (CI: 21–37%). The other common types of malocclusions were crowding (41%, CI: 18–65%), overjet (34%, CI: 21–50%), negative overjet (13%, CI: 7–20%), and crossbite (11%, CI: 7–15%). Additionally, there was no significant difference in Class I (relative risk [RR] = 1.00, [0.96–1.05]), Class II ([RR] = 0.97, [0.92–1.03]), and Class III ([RR] = 1.08, [0.96–1.225]) malocclusion by gender.

**Conclusions:**

This study showed Class I malocclusion has a high prevalence among the Turkish population followed by Class II and Class III malocclusions. In addition, crowding and overjet were the most prevalent malocclusions among Turkish individuals. There were no significant differences in the prevalence of malocclusions between males and females.

## 1 INTRODUCTION

Any variation from the teeth's normal occlusion is what Angle refers to as malocclusion (Sandhu et al., 2012). According to the World Health Organization in 1987, malocclusion is an abnormality that causes disfigurement or affects function and requires treatment if it is an obstacle to the patient's physical or mental well‐being (Sharma et al., 2019). Malocclusions are the third most common oral health issue, behind dental caries and periodontal disorders, (Guo et al., 2016) that significantly affects people's self‐esteem and social acceptance (Balachandran & Janakiram, 2021). Malocclusion must be detected and treated, especially in children, because it affects oral activities like mastication, swallowing and speaking. It also, leads to the development of periodontitis, dental caries, temporomandibular disorders, and trauma (Sanadhya et al., 2014). In addition to enamel defects like Molar‐incisor hypomineralization, (Amrollahi et al., 2023) malocclusion exerts a detrimental influence on oral health‐related quality of life (Baskaradoss et al., 2022).

Malocclusion has a multifactorial etiology and many factors have been attributed to its cause. The main contributing factors are genetic, environmental, and ethnic. Moreover, other causes are the geographical location and the prevalence of malocclusion in the population (Alhammadi et al., 2018). The malocclusion prevalence of Class I, Class II, and Class III was assessed to be 58%, 24%, and 4%, in Denmark (Proffit et al., 1998). On the other hand, the prevalence of Class I malocclusion was between 50 and 55%. The Class II and Class III malocclusion prevalence was 15% and 1%, respectively in the United States (Mills, 1966). Various studies have assessed the prevalence of malocclusion or the Turkish population. While one study showed that the most prevalent malocclusion in Turkish people was Class II division 1 malocclusion, (Gelgör et al., 2007) others demonstrated that Class I malocclusion has the most prevalence (Celikoglu et al., 2010; Gungor et al., 2016; Sayin & Türkkahraman, 2004).

For a multifactorial condition such as malocclusion, accurate epidemiological data is essential. Epidemiology studies, including the prevalence of malocclusion, are beneficial for estimating the size of health issues. It also provides the necessary information and analyzes a potential hypothesis. It establishes the priorities of health programs to plan future actions (Alhammadi et al., 2018; Foster & Menezes, 1976).

Due to the reasons mentioned above, this systematic review and meta‐analysis study aims to generate nationally representative data on individuals' malocclusion in turkey.

## 2 METHODS AND MATERIALS

This systematic review followed the Preferred Reporting Items for Systematic Reviews and Meta‐Analysis Protocol (PRISMA). Registered in PROSPERO with the following ID: CRD42023409379.

The study period was conducted in accordance with PRISMA standards for performing a systematic review (Mehta et al., 2022).

### 2.1 Information sources and literature search

The literature was systematically searched for relevant materials in PubMed/Medline, Web of Science, and Scopus. Data was evaluated through February 2023, with no time restrictions. The studies were searched manually for potential materials. The following search strategies were used for each database, which is shown in Table 1. The included articles were in English and the Turkish language.

**Table 1:** Specific search strategy for each database.

Database	Keyword	Result
PubMed/Medline	(prevalence [Title/Abstract] OR distribution [Title/Abstract] OR epidemiology [Title/Abstract]) AND (malocclusion [Title/Abstract] OR Angle classification [Title/Abstract]) AND (Turkish [All Fields] OR turkey [All Fields] OR Turk [All Fields])	45 papers
Web of Science	prevalence OR distribution OR epidemiology (Topic) and malocclusion OR angle classification (Topic) and turk OR turkish OR turkey (All Fields)	161 papers
Scopus	TITLE‐ABS‐KEY((prevalence OR distribution OR epidemiology) AND (malocclusion OR classification) AND (turkish OR turkey OR turk)) AND (LIMIT‐TO (DOCTYPE,“ar”))	226 papers

Pertinent text words and the medical subject heading (MeSH) were used to build the search strategy. The adopted database search approach is displayed in Table 1.

Relevant studies were searched independently by two authors (M. R. and Q. P.). Abstracts, titles, and entire texts were searched. Before the start of the study, discussion sessions were held to calibrate the reviewers. A list of inclusion and exclusion criteria was prepared and both reviewers were instructed to analyze 22 abstracts. The process was then repeated until a high level of inter‐examiner agreement (*k* = 0.92) was attained. After both reviewers had finished choosing all relevant papers, the results were compared. If there was a discrepancy, a third reviewer (S. H.) was asked to independently evaluate the article for inclusion. After the identification of articles in the database, the articles were imported into the EndNote reference manager for the removal of duplicate articles. In addition, the reference list of retrieved studies was searched to look out for more studies. The relevant study author(s) were contacted via email if any clarifications or extra data were needed.

### 2.2 Study selection (inclusion and exclusion criteria)

This systematic review concentrated on short‐listing the population or school‐based cross‐sectional studies conducted on Turkish individuals. The prevalence of malocclusion according to different indexes and classifications was assessed. The Patient Intervention Comparison Outcome  questions for the present study is as follows:

Patient: Turkish population and ethnicity.

Intervention: None.

Comparison: Different skeletal malocclusion.

Outcome: Prevalence of each skeletal malocclusion.

This review omitted papers that did not describe the prevalence of dental malocclusion, sample size, conference abstracts, case report studies, seminars, case‐control studies, and clinical trials that did not accurately estimate the prevalence. Studies that failed to meet the basic quality evaluation standards were also disqualified.

Studies examining the association between malocclusion and particular factors like nutrition and socioeconomic status, quality of life, caries, periodontal diseases. Also, studies on children with special needs and research on people with any systemic illness or syndrome were excluded from this review.

### 2.3 Data extraction

Study characteristics (study ID, first author's name, year of publication, sampling technique, total sample size in terms of gender and age of the examined population) were retrieved by two independent reviewers (M. R. and Q. P.). Also, exposure description, evaluation (overall and gender‐wise prevalence and severity of malocclusion according to different indices or classification) from eligible studies.

Data was recorded in terms of the percentage of participants falling into each of the following categories for each index or classification:

DAI: no malocclusion (<25), definite malocclusion (26–30), severe malocclusion (31–35) and very severe malocclusion (>35).

Angle's classification: class I, II, III, and normal occlusion

Primary dentition: mesial step, distal step, and flush terminal.

### 2.4 Quality assessment

Two independent reviewers (S. H. and M. D.) qualified the eligible studies for analysis (1.0 Kappa). One researcher (S. H.) was responsible for extracting qualitative or quantitative data from the studies, and the second researcher (M. D.) verified all qualified information. After selecting the articles, assessing the risk of bias in studies was conducted by using a checklist based on the Strengthening the Reporting of Observational Studies in Epidemiology (STROBE) Tool. This checklist contains 12 questions covering various aspects of the methodology such as sample size, study design, sampling method, population, data collection methods, tools, examining sample methods, statistical analysis, and reporting findings based on objectives. Each item corresponded to “yes = 1” or “no = 0”. Therefore, each study was allocated a score of 0−12.

### 2.5 Statistical analysis

Statistical analyses were performed in STATA v.17 software. Heterogeneity between the eligible studies was calculated using Cochrane Q and *I* ^2^ Based on the level of heterogeneity, random or fixed effect models were utilized to estimate the overall prevalence of malocclusion. For each category of indices and classification utilized in the review's scoring criteria. Also, for the overall prevalence, pertinent forest plots were produced, and the confidence interval was maintained at 95%. The data from the included studies were used to create a gender‐specific pooled estimate of prevalence. For publication bias of studies in this meta‐analysis the eager test was performed and the bias was modified using the trim and fill method.

## 3 RESULTS

### 3.1 Search strategy and study quality

**Figure 1:**
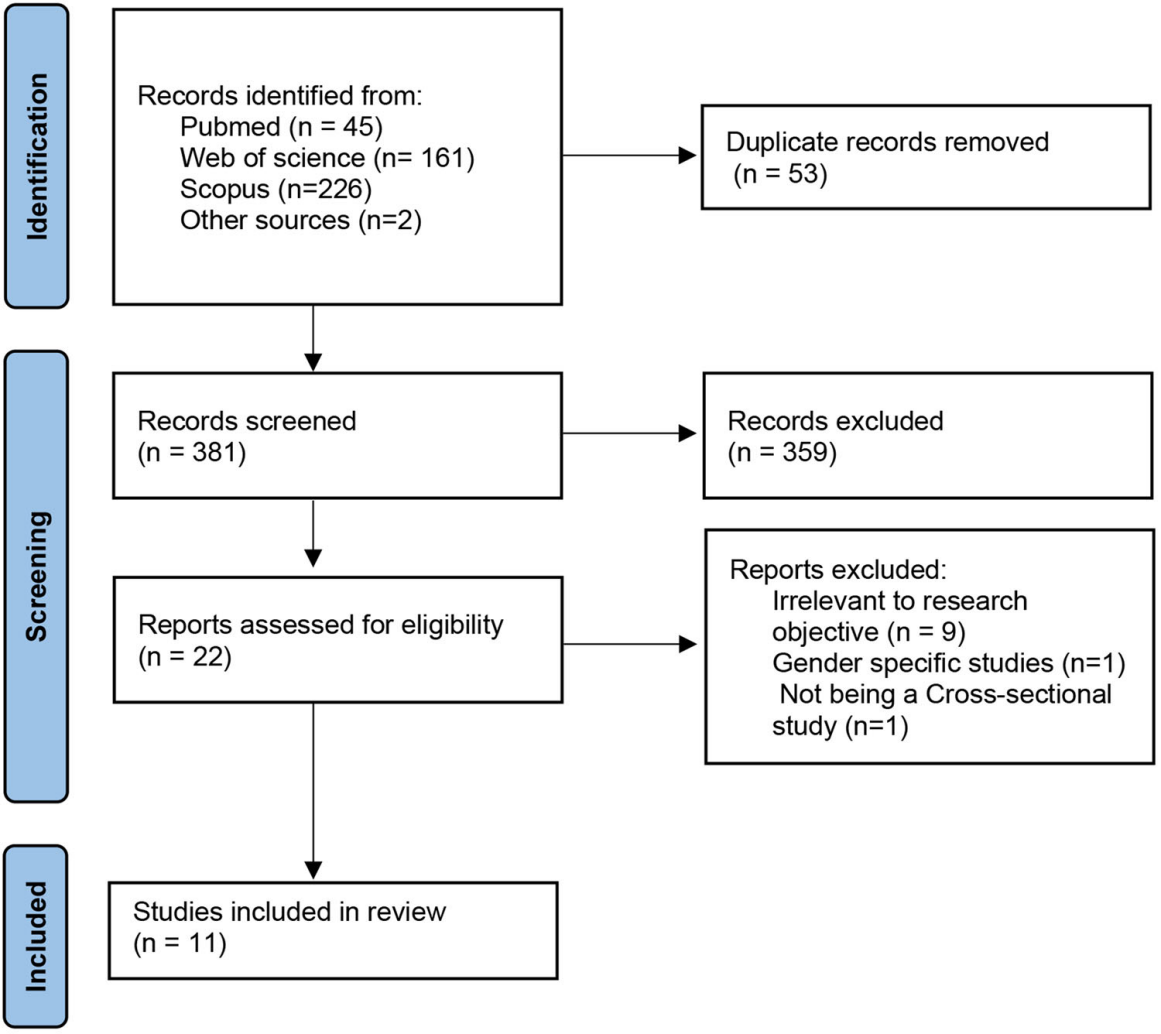
PRISMA flowchart illustrates the included studies' systematic screening/selection process.

The PRISMA flow diagram for the study selection process through different stages is shown in Figure 1, and a completed PRISMA checklist is shown in Appendix A. The initial search yielded 434 results and a total of 381 studies remained after duplication removal. The remaining materials were excluded after reading their titles and abstracts (if necessary). Full texts were retrieved for the remaining papers. Eleven papers were excluded after reading the full text. Figure 1 shows the reason for the exclusion of each paper. The remaining Papers were included in the systematic review and in the meta‐analysis. Based on the STORB appraisal tool (Table 2), the majority of studies had a low risk of bias. However, one study showed a moderate risk of bias, (Akbulut, 2020) and two studies showed a high risk of bias (Kaygisiz et al., 2015; Sari et al., 2003).

**Table 2:** Risk of bias assessments using STROB tool.

	Akbulut (2020)	Uzuner et al. (2015)	Kaygisiz et al. (2015)	Nur et al. (2014)	Celikoglu et al. (2010)	Gelgör et al. (2007)	Yilmaz et al. (2006)	Sayin and Türkkahraman (2004)	Sari et al. (2003)	Başçiftçi et al. (2002)	Uğur (1998)
Are the research questions clearly stated?	Y	Y	Y	Y	Y	Y	Y	Y	Y	Y	Y
Is the approach appropriate for the research question?	Y	Y	Y	Y	Y	Y	Y	Y	Y	Y	Y
Is the study context clearly described?	Y	Y	Y	Y	Y	Y	Y	Y	Y	Y	Y
Is the sampling method clearly described?	N	Y	Y	N	Y	Y	N	Y	N	Y	N
Is the sampling strategy appropriate for the research question?	N	Y	N	Y	Y	Y	Y	Y	N	Y	Y
Is the method of data collection clearly described?	Y	Y	Y	Y	Y	Y	Y	Y	Y	Y	Y
Is the data collection method appropriate for the research question?	Y	Y	Y	Y	Y	Y	Y	Y	Y	Y	Y
Is the method of analysis clearly described?	Y	Y	Y	Y	Y	Y	Y	Y	N	N	Y
Are the main characteristics o the population well described?	Y	Y	Y	Y	Y	Y	Y	Y	Y	Y	Y
Is the method of analysis appropriate for the research question?	Y	Y	N	Y	Y	Y	Y	Y	Y	Y	Y
Are the claims made supported by sufficient evidence?	Y	Y	N	Y	Y	Y	Y	Y	Y	Y	Y
Risk of Bias	M	L	H	L	L	L	L	L	H	L	L

Abbreviations: H, High; L, Low; M, medium; N, no; Y, yes.

### 3.2 Study characteristics

The included studies had diverse age groups ranging from 0 to 38 years old with a total sample size of 13,271 participants. Most of the studies were conducted only on permanent dentition (Akbulut, 2020; Başçiftçi et al., 2002; Celikoglu et al., 2010; Gelgör et al., 2007; Nur et al., 2014; Sayin & Türkkahraman, 2004; Ugur, 1998; Uzuner et al., 2015). However, a few of the studies recruited their sample from universities and dental (nonorthodontic) clinics. Table 3 presents a detailed summary of the main characteristics of each included study.

**Table 3:** Data extraction.

Authors	Year	Sample Size; gender	Age range	Sample source	Malocclusion traits; *n* of cases	Defined I/E criteria	Exam method
Akbulut (2020)	2020	2145; M: 772, F: 1373.	6−29	Firat University, Elazığ	SR: Cl I: 1377, Cl II: 569, Cl III: 199. DR: Cl I: 957, Cl II division 1:792, Cl II division 2: 90, Cl II subdivision: 80, Cl II total: 962, Cl III: 226.	Yes	Clinical and radiography
Uzuner et al. (2015)	2015	457; M:173, F:284.	9−17	Gazi University, Ankara	DR: Cl I:154, Cl II division 1:155, Cl II division 2: 52, Cl II total: 202, Cl III: 96. DAI = 25:95, DAI 26‐30:121, DAI 31‐35: 114, DAI = 35: 127, TL: 93, CR:403, D: 115, OJ:270, NOJ: 129, AOB: 70.	Yes	Clinical
Kaygisiz et al. (2015)	2015	1110; M:549, F: 561.	4.6−23	Gazi University, Ankara	DR: Cl I:853, Cl II:127, Cl III: 130.	Yes	Clinical
Nur et al. (2014)	2014	1023; M:523, F:500.	8−17	Marmara region (*n* = 199), Black Sea region (*n* = 126), East Anatolia region (*n* = 85), Southeastern Anatolia region (*n* = 127), Mediterranean region (*n* = 163), Aegean region (*n* = 213), Central Anatolia region (*n* = 176).	DR: Cl I: 403, Cl II: 495, Cl III: 117 Cl IV (right‐left different molar relations): 4. CR: 208, AOB: 13, LOB: 16, D: 66, AS: 377, C: 6.	Yes	Clinical
Celikoglu et al. (2010)	2010	1507; M: 623, F: 884.	12−25	Turkey	DR: Cl I: 626, Cl II division 1: 435, Cl II division 2: 149, Cl II total: 557, Cl III: 252. CB: 241, OJ: 628, NOJ: 213, AOB: 150, OB:551, CR: 560, D: 68, SB:7.	Yes	Dental cats, Intraoral photographs, and radiography
Gelgör et al. (2007)	2007	2329; M:1125, F: 1204.	12−17	Central Anatolian	DR: Cl I: 812, Cl II division 1: 931, Cl II division 2: 110, Cl II total: 1041, Cl III: 240. OB: 424, AOB:190, OJ: 585, NOJ: 243, CB: 226, SB: 6, CR: 1518, D:164.	Yes	Clinical
Yilmaz et al. (2006)	2006	205; M: 115, F: 90.	3−6	Erzurum province	DR: Cl I: 180, Cl II: 16, Cl III: 9. Flush terminal: 181, Distal step: 15, Mesial step: 9.	Yes	Clinical
Sayin and Türkkahraman (2004)	2004	1356; M:563, F:793.	10−16	Suleyman Demirel University, southern regions of Turkey	DR: Cl I: 875, Cl II division 1: 262, Cl II division 2: 63, Cl II total: 325, Cl III: 156. CR: 233.	Yes	Clinical
Sari et al. (2003)	2003	1602; M:544, F:1058.	0−38	Selcuk university	DR: Cl I:993, Cl II: 449, Cl III: 160. C:22, AOB: 43, D:26, MT: 49.	Yes	Clinical and radiography
Başçiftçi et al. (2002)	2002	965; M: 472, F:493.	6−19	Konya region	DR: Cl I: 737, Cl II division 1: 153, Cl II division 2: 41, Cl II total: 194, Cl III: 34. CB:92, NOJ: 52, CR: 179, OJ: 168, AOB: 79, DB: 211.	Yes	Clinical
Ugur (1998)	1998	572; M: 312, F: 259.	6−10	Ankara, Turkey	TPI: Minor:125, Definite: 143, Sever: 43, Very sever: 29.	Yes	Clinical

Abbreviations: AOB, Anterior open bite; AS, asymmetry, C, cleft; CB, crossbite; CR, Crowding; D, diastema; DAI, dental esthetic index; DB, deep bite; DR, dental relation; F, female; LOB, lateral open bite; M, male; MT, missing teeth; NOJ, negative overjet; OB, overbite; OJ, overjet; SB, scissors bite; SR, skeletal relation; TL, tooth loss; TPI, treatment priority index.

### 3.3 Prevalence of malocclusion traits

The following provides a summary of the malocclusion characteristics found in the studies.

### 3.4 Dental sagittal relation

The overall prevalence of malocclusions in the 10 studies reported the dental relations were 56% for class I, 31% for class II, and 11% for class III malocclusion (Akbulut, 2020; Başçiftçi et al., 2002; Celikoglu et al., 2010; Gelgör et al., 2007; Kaygisiz et al., 2015; Nur et al., 2014; Sari et al., 2003; Sayin & Türkkahraman, 2004; Uzuner et al., 2015; Yilmaz et al., 2006). Furthermore, the remaining 2% were allocated to normal and other types of occlusions such as class IV malocclusion (Celikoglu et al., 2010; Gelgör et al., 2007; Nur et al., 2014; Uzuner et al., 2015). Additional information is mentioned in Table 4. Performing the relative risk meta‐analysis demonstrated that there were no significant differences in malocclusion prevalence between the two genders (Table 5) (Akbulut, 2020; Başçiftçi et al., 2002; Celikoglu et al., 2010; Gelgör et al., 2007; Kaygisiz et al., 2015; Nur et al., 2014). The only study which determined the molar relation of primary dentition showed that 88% of individuals had flash terminal moral relation, 7% had a distal step, and 5% had a mesial step molar relation (Sayin & Türkkahraman, 2004).

**Table 4:** The prevalence of malocclusions.

	Proportion	95% CI	*I* ^2^	Publication bias
Class I	0.56	[0.44−0.68]	99.55%	0.54
Class II	0.31	[0.06−0.42]	99.29%	0.74
Class III	0.11	[0.21−0.37]	97.04%	0.09
Overjet	0.34	[0.21−0.50]	99.17%	0.51
Negative overjet	0.13	[0.07−0.20]	97.91%	0.52
Crossbite	0.11	[0.07−0.15]	94.69%	0.89
Anterior open bite	0.07	[0.03−0.11]	97.543%	0.95
Crowding	0.41	[0.18−0.65]	99.84%	0.384
Diastema	0.04	[0.03−0.04]	99.60%	<0.001^a^

^a^ Modify result by trim and fill method.

**Table 5:** The relative risk of Angel's classifications among different genders.

	Gender	Number of traits	Total number	95% CI	*I* ^2^	Publication bias	*p* Value	Relative risk
Class I	Male	2125	4064	[0.962−1.053]	35.7%	0.83	.784	1.006
	Female	2683	5015					
Class II	Male	1356	4064	[0.924−1.037]	0%	0.75	.467	0.979
	Female	1615	5015					
Class III	Male	413	4064	[0.963−1.224]	0%	0.64	.178	1.086
	Female	559	5015					

### 3.5 Skeletal sagittal relation

Merely one study determined the prevalence of various kinds of skeletal malocclusion in the Turkish population (Akbulut, 2020). Most of the individuals in this study had class I skeletal relation (64%), followed by class II (26%), and (10%) class III relations.

### 3.6 Overjet, negative overjet, open bite, crossbite, crowding, and diastema

The prevalence of overjet and negative overjet were 34% and 13%, respectively (Başçiftçi et al., 2002; Celikoglu et al., 2010; Gelgör et al., 2007; Uzuner et al., 2015). The overall prevalence of crossbite was 11%, (Başçiftçi et al., 2002; Celikoglu et al., 2010; Gelgör et al., 2007) and the frequency of anterior open bite was 7% (Başçiftçi et al., 2002; Celikoglu et al., 2010; Gelgör et al., 2007; Nur et al., 2014; Uzuner et al., 2015). Moreover, six articles reported that the occurrence of crowding and diastemas in the Turkish population was 41% for crowding and 4% for diastemas (Celikoglu et al., 2010; Gelgör et al., 2007; Nur et al., 2014; Sari et al., 2003; Uzuner et al., 2015). There was no study for determining these malocclusion traits among primary dentition. The additional information is mentioned in Table 4.

### 3.7 Dental esthetic index (DAI) and treatment priority index (TPI)

One study used the DAI for the severity of individuals' malocclusion. It showed that 27% of individuals had very severe malocclusion, 24% had severe malocclusion, 26% had definite malocclusion and the rest of the individuals had normal or minor malocclusion (Uzuner et al., 2015). In addition, another study utilized TPI for measuring the severity of individuals' malocclusion. This study demonstrated that the very severe malocclusion was present in 5% of the individuals, 7% of them had severe and 25% definite malocclusion. The remaining 21% had minor malocclusion. Additionally, there were no significant differences among males and females in their malocclusion severity in this study. Even though these two studies used two tools, their outcomes were categorized the same way we calculated the overall outcome of these studies. The pooled prevalence of definite malocclusion was 25%, high17%, and very high severity of malocclusion were and 17%.

## 4 DISCUSSION

People's mental health and oral function may suffer adverse effects from malocclusion (Dimberg, Arnrup, et al., 2015; Magalhães et al., 2010). Determining the prevalence of malocclusion is crucial to organizing, the financial budget and skilled human resources to address the resulting health issues. The current systematic review and meta‐analysis adds critical knowledge to the subject because no previous studies have examined the overall frequency of malocclusion and other teeth‐related defects in the Turkish population at the national level. Class I malocclusion occurred in 56% of the individuals, followed by teeth crowding (41%), overjet (34%), class II malocclusion (31%), negative overjet, (13%), crossbite (11%), class III malocclusion (11%), anterior open bite (7%), and diastemas (4%).

Like other studies that have been conducted on other nationalities such as Iranian, (Akbari et al., 2016) Dutch, (Burgersdijk et al., 1991) Nigerian, Libyans, (Gardiner, 1982) and Egyptian, (El‐Mangoury & Mostafa, 1990) the Turkish population Angel's class I malocclusion has the highest prevalence followed by class II and class III malocclusion. However, two studies that determined the overall prevalence of malocclusion among Saudi Arabian and Chinese individuals showed that class III malocclusion is more prevalent compared to class II malocclusion in these populations (Almotairy & Almutairi, 2022; Shen et al., 2018). The higher predominance of Class III malocclusion in these two populations than others could be attributed to the ethnic differences in craniofacial morphology, genetic predisposition, environmental factors, (Aldrees, 2011; Fleming et al., 2008) and methodological variations such as the participants' age and sample size. Moreover, like the dental relation, the skeletal relation of the two jaws were mainly class I followed by Class II and Class III relations.

There have been controversial results about the occurrence of malocclusion in different populations. Some previous studies reported malocclusions pervasiveness between the two genders were significantly different (Akbari et al., 2016; Lew et al., 1993). On the other hand, the results of the present study indicated that there was no significant difference in malocclusion prevalence between the two genders. In accordance with the present study, the Shen et al. study showed the same result in the Chinese population (Shen et al., 2018). However, due to these controversial outcomes, further gender‐based cohort studies are required to confirm this assumption in the Turkish population.

The major molar relation in Turkish primary dentition was flush terminal molar relation like other studies. The second most common molar relation was a distal step in Turkish children unlike the previous studies which were conducted on Chinese, Indian, and Saudi‐Arabian children (Almotairy & Almutairi, 2022; Lochib et al., 2015; Shen et al., 2018). The results of the current study showed that the majority of children in the Turkish population had a flush terminal molar relation (88%), followed by distal step relations (7%) and mesial step relations (5%). On the other hand, the occurrence of molar relationships in India was flush terminal plane (66.0%), mesial step (12.8%), and distal step (2.4%) (Lochib et al., 2015). The molar position in primary dentition can indicate the future dental relationship of individuals' permanent dentition (Hegde et al., 2012). According to a study by Oneyaso et al. most of cases of flush and mesial terminal plane developed into Angle Class I in the permanent dentition as a result of a combination of forward movement of the mandible and mesial migration of the mandibular arch (Onyeaso & Isiekwe, 2008). However, the frequency of flush terminal relation (88%) among children was higher compared to the incidence of class I malocclusion (56%). Since there was only one study that determined the molar relationship in Turkish children compared to the ten studies on the permanent dentition more studies need to be done.

The (34%) overjet prevalence in the present review was closely associated with the occurrence of (31%) Angle's Class II relationship. Moreover, the overall incidence of Angle's Class III dental relationship (11%) and the negative overjet (13%) also were near each other. The meta‐analysis study on Saudi Arabian population showed the same result regarding the accordance between Class II malocclusion and overjet and the prevalence of Class III and negative overjet malocclusions (Almotairy & Almutairi, 2022). These findings demonstrated that differences between two jaws can lead to Class II and Class III malocclusion which results in an increased or reverse overjet. However, in some cases, the teeth can reduce the relationship between two jaws, especially in Class III individuals.

One study used the DAI, which is advised by the WHO, to standardize epidemiological data on malocclusion and treatment requirements (Organization, 2013). However, the DAI is more of an indicator of the need for esthetic treatment and does not evaluate occlusal factors such as crossbite, asymmetry or impacted teeth (Singh et al., 2011). Furthermore, another study utilized TPI for determining the severity of malocclusions. Although the TPI and DAI both score some orthodontic aspects and evaluate the need for orthodontic treatment, they do not show the incidence of orthodontic features (Jenny & Cons, 1996).

In accordance with previous studies, (Dimberg, Lennartsson, et al., 2015; Proffit et al., 1998) teeth crowding was shown to affect 41% of individuals. The increased frequency of tooth crowding during permanent dentition can be attributed to several factors. Primary teeth serve as the body's natural space maintainers for developing permanent teeth during early childhood. However, if the primary teeth are lost too early or hold on until the eruption of the permanent teeth, the erupting permanent teeth will eventually be pushed aside, leading to crowding of the teeth (Almotairy & Almutairi, 2022).

It is important to realize that studies assessing the prevalence of malocclusion frequently have obvious methodological limitations. These include variations in sample sizes, ages, the source of the sample's recruitment and the process used to register malocclusion traits (Rakhshan, 2013). Determining the occurrence of malocclusion for a particular population using gender‐specific samples or orthodontic patients may not be accurate since these samples are more likely to overstate the presence of malocclusion. This reduces the generalizability of the data. Studies on the prevalence of malocclusions should also consider the fact that different malocclusions are transitory and may disappear or get worse at certain ages. For instance, the occurrence of a midline diastema during the ugly‐duckling stage of the dentition can be corrected by the eruption of permanent canines. As a result, it's important to choose the target age group carefully to prevent any malocclusion feature from being under or overrepresented.

## 5 CONCLUSION

This study showed Class I malocclusion has a high frequency among the Turkish population followed by Class II and Class III malocclusions. In addition, crowding, overjet, negative overjet, and crossbite were the most prevalent malocclusions. There were no significant differences in the occurrence of malocclusions between males and females.

## AUTHOR CONTRIBUTION

**Sara Hashemi**: Corresponding author, oversight, and coordination. **Jimmy Londono and Shohreh Ghasemi**: Principal investigators, conception, and supervision. **Negar Moghaddasi**: Literature search and data extraction. **Homa Baninajarian**: Expertize in the field and supervision. **Amir Fahimipour**: Statistical analysis. **Amirhossein Fathi**: Expertize in the field and supervision. **Mahmood Dashti**: Interpretation and critical revisions. All authors approved the final manuscript for publication.

## CONFLICT OF INTEREST STATEMENT

The authors declare no conflict of interest.

## Data Availability

Data is available through contact with the corresponding author. Email: sara_hsh76@yahoo.com.

## APPENDIX A

**Table:**

Title			
Title	1	Identify the report as a systematic review.	Page 1
Abstract			
Abstract	2	See the PRISMA 2020 for Abstracts checklist.	Done
Introduction			
Rationale	3	Describe the rationale for the review in the context of existing knowledge.	Page 2
Objectives	4	Provide an explicit statement of the objective(s) or question(s) the review addresses.	Page 2
Methods			
Eligibility criteria	5	Specify the inclusion and exclusion criteria for the review and how studies were grouped for the syntheses.	Page 3,4
Information sources	6	Specify all databases, registers, websites, organizations, reference lists and other sources searched or consulted to identify studies. Specify the date when each source was last searched or consulted.	Page 3
Search strategy	7	Present the full search strategies for all databases, registers and websites, including any filters and limits used.	Page 3,15, Table 1
Selection process	8	Specify the methods used to decide whether a study met the inclusion criteria of the review, including how many reviewers screened each record and each report retrieved, whether they worked independently, and if applicable, details of automation tools used in the process.	Page 3,4
Data collection process	9	Specify the methods used to collect data from reports, including how many reviewers collected data from each report, whether they worked independently, any processes for obtaining or confirming data from study investigators, and if applicable, details of automation tools used in the process.	Page 3
Data items	10a	List and define all outcomes for which data were sought. Specify whether all results that were compatible with each outcome domain in each study were sought (e.g., for all measures, time points, analyses), and if not, the methods used to decide which results to collect.	Page 4
	10b	List and define all other variables for which data were sought (e.g., participant and intervention characteristics, funding sources). Describe any assumptions made about any missing or unclear information.	Page 3,4
Study risk of bias assessment	11	Specify the methods used to assess risk of bias in the included studies, including details of the tool(s) used, how many reviewers assessed each study and whether they worked independently, and if applicable, details of automation tools used in the process.	Page 4
Effect measures	12	Specify for each outcome the effect measure(s) (e.g., risk ratio, mean difference) used in the synthesis or presentation of results.	Page 5
Synthesis methods	13a	Describe the processes used to decide which studies were eligible for each synthesis (e.g., tabulating the study intervention characteristics and comparing against the planned groups for each synthesis (item #5)).	Page 3,4
	13b	Describe any methods required to prepare the data for presentation or synthesis, such as handling of missing summary statistics, or data conversions.	
	13c	Describe any methods used to tabulate or visually display results of individual studies and syntheses.	
	13d	Describe any methods used to synthesize results and provide a rationale for the choice(s). If meta‐analysis was performed, describe the model(s), method(s) to identify the presence and extent of statistical heterogeneity, and software package(s) used.	Page 5
	13e	Describe any methods used to explore possible causes of heterogeneity among study results (e.g., subgroup analysis, meta‐regression).	Page 5
	13f	Describe any sensitivity analyses conducted to assess robustness of the synthesized results.	
Reporting bias assessment	14	Describe any methods used to assess risk of bias due to missing results in a synthesis (arising from reporting biases).	Page 4
Certainty assessment	15	Describe any methods used to assess certainty (or confidence) in the body of evidence for an outcome.	Page 5
Results			
Study selection	16a	Describe the results of the search and selection process, from the number of records identified in the search to the number of studies included in the review, ideally using a flow diagram.	Page 6, Page 14, Figure 01
	16b	Cite studies that might appear to meet the inclusion criteria, but which were excluded, and explain why they were excluded.	Page 6, Page 14, Figure 01
Study characteristics	17	Cite each included study and present its characteristics.	Page 6, Page 17,18, Table 3
Risk of bias in studies	18	Present assessments of risk of bias for each included study.	Page 6, Page 16, Table 2
Results of individual studies	19	For all outcomes, present, for each study: (a) summary statistics for each group (where appropriate) and (b) an effect estimate and its precision (e.g., confidence/credible interval), ideally using structured tables or plots.	Page 6,7, Page 17,18, Table 3
Results of syntheses	20a	For each synthesis, briefly summarize the characteristics and risk of bias among contributing studies.	Page 16, Table 2
	20b	Present results of all statistical syntheses conducted. If meta‐analysis was done, present for each the summary estimate and its precision (e.g., confidence/credible interval) and measures of statistical heterogeneity. If comparing groups, describe the direction of the effect.	Page 19,20, Table 4,5
	20c	Present results of all investigations of possible causes of heterogeneity among study results.	
	20d	Present results of all sensitivity analyses conducted to assess the robustness of the synthesized results.	
Reporting biases	21	Present assessments of risk of bias due to missing results (arising from reporting biases) for each synthesis assessed.	Page 16, Table 2
Certainty of evidence	22	Present assessments of certainty (or confidence) in the body of evidence for each outcome assessed.	
Discussion			
Discussion	23a	Provide a general interpretation of the results in the context of other evidence.	Page 8,9
	23b	Discuss any limitations of the evidence included in the review.	Page 10
	23c	Discuss any limitations of the review processes used.	
	23d	Discuss implications of the results for practice, policy, and future research.	Page 10
Other information			
Registration and protocol	24a	Provide registration information for the review, including register name and registration number, or state that the review was not registered.	
	24b	Indicate where the review protocol can be accessed, or state that a protocol was not prepared.	
	24c	Describe and explain any amendments to information provided at registration or in the protocol.	
Support	25	Describe sources of financial or nonfinancial support for the review, and the role of the funders or sponsors in the review.	None
Competing interests	26	Declare any competing interests of review authors.	None
Availability of data, code and other materials	27	Report which of the following are publicly available and where they can be found: template data collection forms; data extracted from included studies; data used for all analyses; analytic code; any other materials used in the review.	

*From:* Page MJ, McKenzie JE, Bossuyt PM, Boutron I, Hoffmann TC, Mulrow CD, et al. The PRISMA 2020 statement: an updated guideline for reporting systematic reviews. BMJ 2021;372:n71. doi: 10.1136/bmj.n71 For more information, visit: http://www.prisma-statement.org/
